# Uterine artery embolization or myomectomy for women with uterine fibroids: Four-year follow-up of a randomised controlled trial

**DOI:** 10.1016/j.eurox.2021.100139

**Published:** 2021-11-20

**Authors:** Jane Daniels, Lee J. Middleton, Versha Cheed, William McKinnon, Fusun Sirkeci, Isaac Manyonda, Anna-Maria Belli, Mary Ann Lumsden, Jonathan Moss, Olivia Wu, Klim McPherson, on behalf of the FEMME Trial Collaborative Group

**Affiliations:** a Nottingham Clinical Trials Unit, University of Nottingham, Nottingham NG7 2RD, UK; b Birmingham Clinical Trials Unit, University of Birmingham, Birmingham B15 2TT, UK; c Department of Obstetrics and Gynaecology, Whipps Cross Hospital, London E11 1NR, UK; d Department of Gynaecology, St George’s Hospital and Medical School, London SW17 0QT, UK; e Department of Radiology, St George’s Hospital and Medical School, London SW17 0QT, UK; f School of Medicine, University of Glasgow, Glasgow G31 2ER, UK; g Institute of Health and Wellbeing, University of Glasgow, Glasgow G12 8RZ, UK; h Department of Primary Care, University of Oxford, OX3 9DU, UK

**Keywords:** UAE, uterine artery embolization, UFS-QoL, Uterine Fibroid Scale -Quality of Life, PBAC, Pictorial Blood loss Assessment Chart, Adult, Female, Human, Myomectomy, Pregnancy rate, Quality of life, Randomised controlled trial, Uterine fibroid, Uterine artery embolization

## Abstract

**Objective:**

To examine the quality of life experienced by women with symptomatic uterine fibroids who had been treated with UAE in comparison to myomectomy. We report the four-year follow-up of the FEMME randomised trial. Two-year follow-up data has been previously reported.

**Study Design:**

Premenopausal women who had symptomatic uterine fibroids amenable to myomectomy or uterine artery embolization were recruited from 29 UK hospitals. Women were excluded if they had significant adenomyosis, any malignancy, pelvic inflammatory disease or had had a previous open myomectomy or uterine artery embolization.

Participants were randomised to myomectomy or embolization in a 1:1 ratio using a minimisation algorithm. Myomectomy could be open abdominal, laparoscopic or hysteroscopic, according to clinician preference. Embolization of the uterine arteries was performed according to local practice, under fluoroscopic guidance.

The primary outcome measure was the Uterine Fibroid Symptom Quality of Life questionnaire, adjusted for baseline score and reported here at four years post-randomisation. Subsequent procedures for fibroids, pregnancy and outcome were amongst secondary outcomes.

Trial registration ISRCTN70772394 https://doi.org/10.1186/ISRCTN70772394

**Results:**

254 women were randomized, 127 to myomectomy (105 underwent myomectomy) and 127 to uterine artery embolization (98 underwent embolization). At four years, 67 (53%) and 81 (64%) completed UFS-QoL quality of life scores. Mean difference in the UFS-QoL at 4 years was 5.0 points (95% CI −1.4 to 11.5; p = 0.13) in favour of myomectomy. There were 15 pregnancies in the UAE group and 7 in the myomectomy group, with a cumulative pregnancy rate to four years of 15% and 6% respectively (hazard ratio: 0.48; 95% CI 0.18–1.28). The cumulative repeat procedure rate to four years was 24% in the UAE group and 13% in the myomectomy group (hazard ratio: 0.53; 95% CI 0.27–1.05).

**Conclusions:**

Myomectomy resulted in greater improvement in quality of life compared with uterine artery embolization, although by four years, this difference was not statistically significant. Missing data may limit the generalisability of this result. The numbers of women becoming pregnant were too small draw a conclusion on the effect of the procedures on fertility.

## Introduction

Uterine fibroids are the most common tumour in women of reproductive age, and are associated with heavy menstrual bleeding, abdominal discomfort, subfertility, and reduced quality of life. The levonorgestrel-releasing intra-uterine device is the first line treatment for small (<3 cm) fibroids, [1] whilst the future for ulipristal acetate as long term treatment is uncertain. [2].

Surgery, either myomectomy or hysterectomy, has traditionally been the main approach for management of symptomatic fibroids. Uterine artery embolization (UAE) involves temporary occlusion of the arteries supplying the uterus using biocompatible particles and is usually performed under local anaesthetic.

For women seeking to keep their uterus, there was limited information on the relative effectiveness of myomectomy and UAE to guide their decision. Before the FEMME trial, there had been two previous randomized trials comparing UAE and myomectomy, [1], [2], [3] involving a total of 242 women, with substantial attrition in one, [1] and follow-up only to two years post-randomisation. These trials did not report on reproductive outcomes. [4].

We performed the multicentre, randomised trial treating Fibroids with Embolization or Myomectomy to Measure the Effect on quality of life among women wishing to avoid hysterectomy (the FEMME study). We have reported on the primary outcome of condition-specific health-related quality of life (HRQoL) using the Uterine Fibroid Symptom Quality of Life questionnaire at two years. [5] Average HRQoL scores at two years were substantially improved in both groups, but these improvements were greater in those assigned to myomectomy (mean difference 8.0 points; 95%CI: 1.8, 14.1; p = 0.01). These differences were robust to sensitivity analyses and no differential effect was seen in any subgroups. Menstrual bleeding scores appeared similar in both groups, although other participant reported secondary outcomes (symptom severity, general quality of life and acceptability) saw greater improvements with myomectomy. Ovarian reserve, defined by levels of follicle stimulating hormone, anti-Mullerian hormone and luteinising hormone, at 6 weeks, 6 months and 1-year post-procedure did not demonstrate a material difference between the groups. Peri- and post-operative complications from all initial procedures occurred in similar percentages of women in both groups.

We continued the trial until all participants had reached the four-year post-procedure timepoint, collecting the same participant reported outcomes as at two years from all participants except those who had withdrawn consent, and sought information on pregnancy and pregnancy outcomes, and further procedures for fibroids.

## Methods

The FEMME trial was a randomised, open, parallel multi-centre trial and details of the protocol have been previously published. [6] Women were eligible for the study if they were> 18 years of age, pre-menopausal and were not pregnant. Women were excluded if they had significant adenomyosis, suspected or diagnosed malignancy, recent or ongoing pelvic inflammatory disease or had had a previous open myomectomy or uterine artery embolization. All participants gave written informed consent for trial participation. A gynaecologist diagnosed uterine fibroids following history taking, pelvic examination and an ultrasound scan, and determined whether the fibroid was amenable to myomectomy. An interventional radiologist assessed whether uterine artery embolization was feasible, usually by seeing the patient and performing a contrast enhanced magnetic resonance scan. Women were only eligible if either procedure was feasible and appropriate. Due to the nature of the procedures, all participants, clinicians, and trial nurses were aware of the group allocations.

Participants were randomly assigned in a 1:1 ratio to undergo myomectomy or uterine artery embolization as the initial procedure. Randomization was performed centrally through a secure internet facility once the eligible woman had provided informed consent. A minimization algorithm balanced the study-group allocations according to longest dimension of largest fibroid (≤7 cm or>7 cm), number of fibroids (1–3, 4–10,>10), and if the woman desired pregnancy.

Myomectomy was via the route preferred by the operating gynaecologist, either as an open, hysteroscopic, laparoscopic or combination procedure. Ulipristal acetate or gonadotrophin releasing hormone analogue were used pre-operatively if considered essential by the gynaecologist. Concurrent procedures, for example adhesiolysis, were not restricted.

Bilateral selective catheterization and embolization of the uterine arteries was performed by the interventional radiologist under fluoroscopic guidance. The embolic agent and angiographic endpoint were at the discretion of the interventional radiologist.

The original primary outcome measure was repeated at the four-year timepoint and was the condition-specific quality of life (HRQoL) domain score from the UFS-QOL questionnaire. Scores range from zero indicating worst score to 100, higher scores indicating better health-related quality of life. The instrument demonstrates face, construct and discrimination validity and is responsive to change. [7], [8], [9] Pre-specified secondary outcomes were, symptom severity domain from the UFS-QoL (scores range from zero [no symptoms] to 100 [worst symptoms]); EuroQoL EQ‐5D‐3L score (on the scale −0.59 [worst] to 1.0 [perfect]), [10] and the EuroQoL health thermometer score (on the scale 0 [worst] to 100 [perfect]). Menstrual blood loss was estimated by the Pictorial Blood loss Assessment Chart (PBAC), (scores range from 0 [no bleeding] as a minimum but have no fixed upper limit) [11]. The PBAC was also used to generate rates of amenorrhea and non-heavy bleeding. Other secondary outcomes were pregnancy, the time interval to the first pregnancy and its outcome (live birth, miscarriage, still birth and termination; overall and in the population desiring pregnancy at the time of randomization); participant satisfaction (determined by questions “would you have your operation again?” and “would you recommend operation to a friend?”), and time to further fibroid treatment. A questionnaire booklet was posted to all participants except those who had withdrawn consent to be followed-up, and non-responders recontacted. Our outcomes mapped to a core outcome set for trials in women with fibroids. [12].

A sample size of 250 had 90% power to detect a moderate-sized difference between groups (0.55 of a standard deviation) at two years, the primary timepoint for the trial. An explanation for a mid-trial revision of the sample size is given in the main trial publication. [5].

Participants remained in the group to which they were allocated, regardless of subsequent treatments and analysis performed according to the intention-to-treat principle. All participant reported questionnaires producing continuous measures used a repeated measures linear regression model, [13] including data at all time points, to estimate mean differences and 95% two-sided confidence intervals at the time of four years. Parameters allowing for participant, treatment group, time, time by treatment interaction and the minimization variables were included as fixed effects. Participants were included in the model provided they had at least one response at any of the four assessment times and incomplete responses were assumed to be missing at random. Log-binomial regression was used to estimate relative rates and 95% confidence intervals for binary outcomes, making similar adjustments to the continuous outcome analyses.

## Results

### Trial participants

**Fig. 1:**
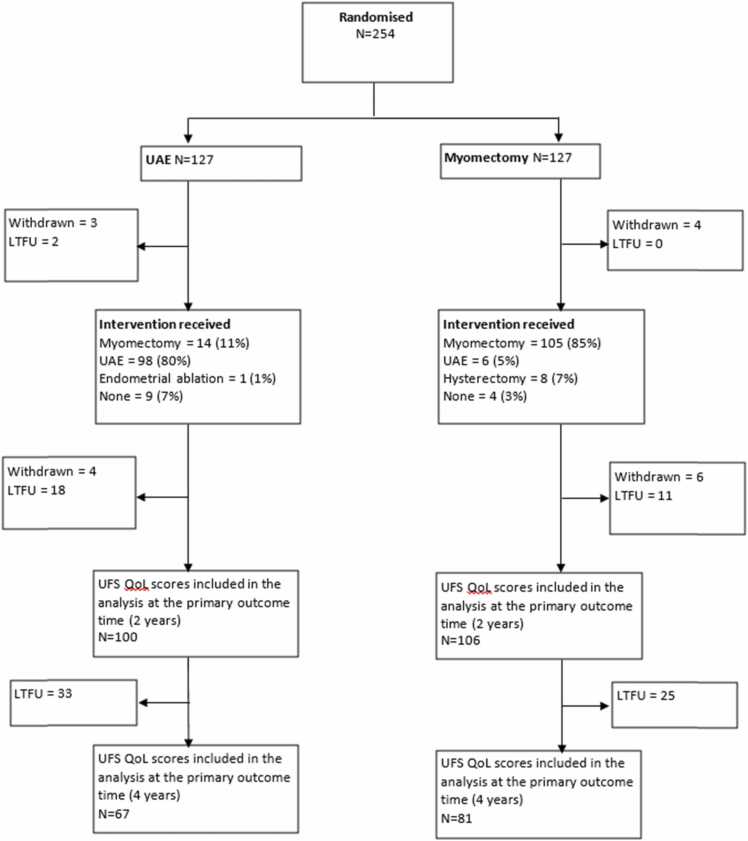
Patient flow through the FEMME trial to four-year follow-up.

Randomisation of participants from 29 UK hospitals took place between 6th February 2012 and 21st May 2015. A total of 127 participants were assigned to myomectomy and 127 to UAE, shown in Fig. 1. Of the 123 women randomised to myomectomy and did not withdraw from the trial prior to procedure, 105 (85%) had a myomectomy as their initial operation. Similarly, 98 of the 122 (80%) women in the UAE group, underwent UAE. The baseline characteristics are shown in Table 1. Women were, on average 41 years old, classed as overweight by their body mass index, and 48% of participants in both groups were seeking to get pregnant.

**Table 1:** Baseline demographic, medical, surgical and fibroid characteristics of all trial participants.

		Uterine Artery Embolization (n = 127)	Myomectomy (n = 127)
**Demographics and obstetric history**			
Age, years	Mean (SD)	40.2 (6.55), 127	42.7 (6.4), 127
Ethnic Group	White (British/Other)	59 (46%)	57 (45%)
	Black (Caribbean/African/other)	48 (38%)	54 (43%)
	South Asian (Indian/Pakistani/Bangladeshi)	10 (8%)	5 (4%)
	Mixed (White/Black/Asian/other)	6 (5%)	8 (6%)
	Other	4 (3%)	3 (2%)
BMI, kg/m^2^	Mean (SD), n	28.2 (6.2), 119	28.1 (5.3), 123
Desiring pregnancy at time of randomization		61 (48%)	61 (48%)
Parity	Median [IQR], n	0 [0,1], 125	1 [0,2], 127
Gravida	Median [IQR], n	1 [0,2], 125	2 [0,3], 127
**Fibroid assessment**			
Scanning modality to diagnose fibroid^b^	MRI	89 (71%)	99 (79%)
	Ultrasound	36 (29%)	27 (21%)
	Not stated	2	1
Location of largest fibroid	Submucosal	6 (5%)	14 (11%)
	Submucosal (pedunculated)	1 (1%)	1 (1%)
	Subserosal	30 (25%)	21 (17%)
	Subserosal (pedunculated)	6 (5%)	5 (4%)
	Intramural	74 (61%)	81 (64%)
	Other	4 (3%)	0 (-)
	Not stated	6	5
Longest dimension of largest fibroid, cm^a^	<=7	64 (50%)	64 (50%)
	>7	63 (50%)	63 (50%)
	Mean (SD)	7.6 (3.2)	7.7 (4.2)
Number of fibroids^a^	1–3	84 (66%)	84 (66%)
	4–10	37 (29%)	37 (29%)
	>10	6 (5%)	6 (5%)
	Median [IQR]	2 [1,5]	2 [1,5]
Largest fibroid volume, cm^3^	Mean (SD), n	436 (594), 124	446 (548), 126
Uterine volume, cm^3^	Mean (SD), n	1170 (1280), 118	1240 (1120), 118
**Medical and surgical history**			
Previous abdominal surgery^c^	Caesarean section	12 (9%)	19 (15%)
	Laparoscopy	19 (15%)	15 (12%)
	Endometrial ablation	3 (2%)	2 (2%)
	Appendectomy	8 (6%)	7 (6%)
	Sterilization	4 (3%)	5 (4%)
	Other	10 (8%)	15 (12%)
Taking contraceptive/ hormonal treatments to control symptoms, at randomization		75 (59%)	73 (57%)

a Minimisation variable;

b More than one type of scan possible;

c More than one previous abdominal surgery possible. Table reproduced from Manyonda 2020.

Procedural details have been published previously, but briefly, in the UAE group, radiologists judged a complete or near complete infarction of the fibroids in 57 of 80 (77%) women who had a MRI scan at 6 months post-procedure. In the myomectomy group, 93 of 105 (89%) women had an open abdominal myomectomy. Peri and post-operative complications occurred in 27 of 113 (24%) women in the UAE group and 34 of 118 (29%) in the myomectomy group, with a relative risk of 1.2 (95%CI 0.8–1.9; p = 0.4).

At four years post-randomisation, 67 (53%) and 81 (64%) women returned complete UFS-QoL quality of life scores. The baseline characteristics of the responders are shown in Table S1. The mean health related quality of life score was 86.6 (standard deviation [SD] 20.5) in the UAE group and 90.2 (SD 19.7) in the myomectomy group, a mean difference of 5.0 (95% confidence interval [CI] −1.4 to 11.5). The mean severity score was 18.8 (SD 18.8, n = 70) in the UAE group and 14.5 (SD 17.5, n = 80) in the myomectomy group, mean difference − 5.0 (95% CI −10.8 to, 0.8). The EQ-5D score was 0.79 (SD 0.30. n = 70) in the UAE group and 0.90 (SD0.16, n = 83) in the myomectomy group, mean difference 0.13 (95% CI 0.06–0.20). The Health Thermometer score was 75.3 (SD 19.4, n = 71) in the UAE group and 82.8 (SD 17.5, n = 82) in the myomectomy group, mean difference 8.7 (95%CI 3.5–13.8).

There was no evidence of any differences in menstrual bleeding between the groups (Table 2), with the majority of menstruating women reporting regular or fairly regular periods, 36 of 48 (75%) in the UAE group, 30 of 39 (77%) in the myomectomy group. There were no apparent differences in the participants’ rating of their operation by four years, which remained high overall. In the UAE group, 50 of 66 (76%) women would choose their operation again, and 51 of 64 (80%) would recommend it to a friend, compared to 59 of 78 (76%) and 69 of 76 (91%), respectively, in the myomectomy group.

**Table 2:** Pictorial Blood Assessment Chart bleeding scores and categories within four years.

PBAC score or category	UAE	Myomectomy	
	Median [IQR]	**Median [IQR]**	
Total score	28 [0–75]	29 [0–81]	
	**Mean (SD)**	**Mean (SD)**	**Estimated Relative risk; 95%CI**
Total score (log-transformed)^a^	2.8 (2.0)	2.6 (2.0)	-0.01 (−0.4, 0.4)
Amenorrhea (=0)	14 (27%)	15 (35%)	1.3 (0.7, 2.3)^b^
Light (1–10)	3 (6%)	1 (2%)	
Normal (>10–100)	26 (51%)	21 (49%)	
Heavy (>100)	8 (16%)	6 (14%)	0.9 (0.4, 2.4)
**Total**	**51**	**43**	

a In order for PBAC scores of 0 to be included for log transformed scores, all responses have been transformed by adding 1 then taking the log.

b Un-adjusted model used as adjusted model failed to converge

There were 15 pregnancies reported by 12 women in the UAE group, and six pregnancies amongst seven women in the myomectomy group. The outcomes of the pregnancies within four years, and rates according to per-protocol and treatment received groups, are shown in Table 3. The cumulative pregnancy rate was 15% in the UAE group and 6% in the myomectomy group (hazard ratio from intention-to-treat data: 0.48; 95% CI 0.18–1.28).

**Table 3:** Pregnancy outcomes within four years.

	UAENumber of women (number of events)	Myomectomy Number of women (number of events)
**Pregnancy by Intention to Treat**		
Women reporting pregnancy^a^	12 (15)	6 (7)
Pregnancy (in population desiring pregnancy at time of randomisation)	12 (15)	6 (7)
Live birth	7 (9)	5 (6)
Miscarriage	4 (5)	0
Termination	1	1
**Pregnancy by Per Protocol**		
Women reporting pregnancy^b^	7 (8)	6 (7)
Live birth	4 (5)	5 (6)
Miscarriage	2	0
Termination	1	1
**Pregnancy by treatment received**		
Women reporting pregnancy^c^	7 (8)	8 (10)
Live birth	4 (5)	6 (7)
Miscarriage	2	1 (2)
Termination	1	1

a UAE Group: One participant had two pregnancies, both ending in miscarriage; two participants had two pregnancies, both ending in live birth; Myomectomy Group: One participant had two pregnancies, both ending in live birth. These events have been primarily included once in this table, with repeat events in the same woman shown in brackets. All other events occurred in separate women. Percentages of the total population cannot be calculated as women withdrew or were lost from follow-up to the trial at different intervals up to four years.

b UAE Group: One participant had two pregnancies, both ending in live birth; Myomectomy Group: One participant had two pregnancies, both ending in live birth. These events have been primarily included once in this table, with repeat events in the same woman shown in brackets. All other events occurred in separate women. Percentages of the total population cannot be calculated as women withdrew or were lost from follow-up to the trial at different intervals up to four years.

c UAE Group: One participant had two pregnancies, both ending in live birth; Myomectomy Group: One participant had two pregnancies, both ending in live birth; One participant had two pregnancies, both ending in miscarriage. These events have been primarily included once in this table, with repeat events in the same woman shown in brackets. All other events occurred in separate women. Percentages of the total population cannot be calculated as women withdrew or were lost from follow-up to the trial at different intervals up to four years

**Fig. 2:**
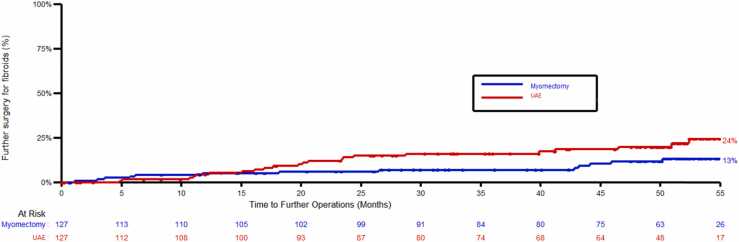
Time to further procedure for fibroids. Footnote. Operation dates were estimated to be halfway between follow-up times if exact dates were not available.

The cumulative number of further procedures for treatment of fibroids was 22 in the UAE group and 13 in the myomectomy group, which were hysterectomies for 11 and 8 women, myomectomy for 6 and 2 women and transcervical resection for 5 and 3 women, respectively. Percentages are not presented here due to the high levels of drop out by 4 years preclude an appropriate denominator. The cumulative repeat procedure rate was 24% in the UAE group and 13% in the myomectomy group (hazard ratio: 0.53; 95% CI 0.27–1.05), shown in Fig. 2.

## Discussion

### Main findings

Both procedures improved participant reported health-related quality of life scores, and by four years, women assigned myomectomy still reported marginally higher scores than those in the UAE group, but the difference was no longer statistically significant, as it was at two years. Menstrual bleeding scores appeared similar in both groups. The need for additional treatments was higher in the UAE group. There were 15 pregnancies in the UAE group and 7 in the myomectomy group in total.

### Strengths and limitations

This study is the largest ever randomised clinical trial to report on treatment of symptomatic fibroids by UAE and myomectomy and has the longest follow-up of participants. Randomisation via computer-generated allocation sequence was effective in achieving balanced groups with respect to important prognostic factors. The outcomes reflect the aspects of the condition that are most important to women. The broad eligibility criteria, substantial proportion of women of African-Caribbean ethnicity and inclusion of women regardless of their pregnancy intentions reflects the diversity of fibroid presentations. The multicentred recruitment allowed both procedures’ impact to be assessed without confounding by individual clinicians’ preferences and skill.

As with all longer-term studies using participant-reported data, the quantity of data decreases over time, with 42% of UFS-QOL data missing at four years, an increase from 19% missing UFS-QOL data at two years post-randomisation, limiting the generalisability of the results. The number of women reporting menstrual bleeding data is further decreased by women entering the perimenopausal state. We did not use multiple imputation for missing data for this analysis. The greater number of women having repeat procedures for their fibroid symptoms in the UAE group will impact on the participant-reported outcomes, which could be expected to improve again. Finally, there are too few women attempting to become pregnant, partly as a consequence of the average age at randomisation, for any meaningful interpretation of the pregnancy data.

### Interpretation

The between group difference of 8 points (95%CI 1.8–14.1) on the health-related quality of life domain seen within two-years reduced to 5 points by four years, representing a very small standardised effect size which is unlikely to be clinically significant. This may be unsurprising given the reasonable proportions of women who, by 4 years after treatment allocation, had either had a subsequent procedure that worked for them, or had ceased menstruating either naturally or through surgical intervention.

There are no other longer-term data from randomised controlled trials of UAE compared with myomectomy. Meta-analysis of the two studies comparing UAE with hysterectomy or myomectomy did not show a difference in the number of repeat interventions (pooled risk ratio 3.45, 95%CI 0.18 – 64.35; p = 0.45, I^2^ =75%) within two years, and no evidence of a difference in the proportion of women who would recommend their procedure after 5 years (RR 1.11, 95%CI 0.94 – 1.32; p = 0.16, I^2^ =50%). [14].

Women desiring pregnancy should be provided with the evidence generated from the FEMME trial, for although the impact of myomectomy and UAE on fertility remains uncertain, there was no evidence of any material difference between the levels of hormones associated with ovarian reserve in each group at 12 months post-procedure.

A cost-utility assessment of the two procedures based on the FEMME trial has been published elsewhere. [15] UAE is dominated by myomectomy and would not be considered a cost-effective alternative over myomectomy, but this does not take into account any potential preference for a less invasive procedure. There is such a small difference in costs between the two procedures, fully informed patient preference should be acknowledged, and women should have the option to choose between the two procedures.

## Conclusion

In conclusion, both UAE and myomectomy are effective treatments for improving the quality of life of women with symptomatic uterine fibroids, but the advantage of myomectomy observed is not sustained at four years. Both procedures should be continued to be offered to women, including those desiring a future pregnancy.

The FEMME trial provides high quality evidence on the clinical effectiveness of UAE compared to myomectomy in the medium and long-term. Further clinical trials addressing this pragmatic question around quality of life are not required, but long-term follow-up of the FEMME cohort using routine data to capture further reinterventions would be of benefit. The impact of myomectomy compared to UAE on live birth rates remains unanswered.

## Details of ethics approval

The trial had a favourable ethical opinion from National Research Ethics Service (NRES) Committee Coventry and Warwickshire (11/WM/0149).

## FUNDING

National Institute of Health Research (NIHR) Health Technology Assessment programme reference number 08/53/22. A monograph reporting the data collected in this trial has been submitted for publication in the NIHR Journals Library. Further information is available at www.journalslibrary.nihr.ac.uk/hta. The views and opinions expressed by authors in this article are those of the authors and do not necessarily reflect those of the National Health Service, the NIHR, the NIHR Central Commissioning Facility, the NIHR Evaluation, Trials and Studies Coordinating Centre, the NIHR Health Technology Assessment program, or the Department of Health and Social Care.

## CRediT authorship contribution statement

**JD:** conceptualization, writing (original draft,), writing (review and editing), project administration, investigation, funding acquisition. **LM:** statistical analysis plan, data curation, formal analysis, writing (review and editing). **VC:** data curation, formal analysis, writing (review and editing). **WM:** writing (review and editing), investigation, project administration. **FS:** investigation, writing (review and editing). **IM:** conceptualization, site principal investigator, writing (review and editing), investigation, funding acquisition. **A-MB:** conceptualization, site principal investigator, writing (review and editing), investigation, funding acquisition. **M-AL:** conceptualization, site principal investigator, writing (review and editing), investigation, funding acquisition. **JM:** conceptualization, site principal investigator, writing (review and editing), investigation, funding acquisition. **OW:** conceptualization, writing (review and editing), funding acquisition. **KM:** conceptualization, statistical analysis plan, writing (review and editing), funding acquisition.

## Trial oversight

Study oversight and monitoring were provided by a Trial Steering Committee and by an independent Data Monitoring Committee.

The TSC provided independent supervision for the trial, providing advice to the chief and co-investigators and the sponsor on all aspects of the trial throughout the trial. The DMC adopted the DAMOCLES charter to define its terms of reference and operation in relation to oversight of the FEMME trial. Both committees met on an approximately annual basis during the period of recruitment and follow-up.

## Disclosure of interests

MAL reports personal fees from Gedeon Richter outside the submitted work.

All other authors declare they have no competing interests.

All authors have completed the unified competing interest form at www.icmje.org/coi_disclosure.pdf (available in the support information).
