# Supplementary material for: Uterine artery embolization or myomectomy for women with uterine fibroids: Four-year follow-up of a randomised controlled trial
Source: Eur J Obstet Gynecol Reprod Biol X. 2021 Nov 20;13:100139. doi: 10.1016/j.eurox.2021.100139 (PMC8633559; doi:10.1016/j.eurox.2021.100139)
Supplement: Supplementary file 1 — Supplementary material [file mmc1.docx]

|  |  | **Uterine Artery Embolization** (n=67) | **Myomectomy** (n=81) | **p-value^d^** |
| --- | --- | --- | --- | --- |
| **Demographics and obstetric history** | | | |  |
| Age, years | Mean (SD) | 41.0 (6.6), 67 | 43.2 (6.3), 81 | 0.04 |
| Ethnic Group | White (British/Other) | 38 (57%) | 37 (46%) | 0.77 |
|  | Black (Caribbean/African/other) | 22 (33%) | 33 (41%) |  |
|  | South Asian (Indian/Pakistani/Bangladeshi) | 3 (4%) | 3 (4%) |  |
|  | Mixed (White/Black/Asian/other) | 3 (4%) | 5 (6%) |  |
|  | Other | 1 (1%) | 3 (4%) |  |
| BMI, kg/m^2^ | Mean (SD), n | 27.7 (5.0), 63 | 27.8 (5.0), 77 | 0.98 |
| Desiring pregnancy at time of randomization^a^ | | 27 (40%) | 36 (44%) |  |
| Parity | Median [IQR], n | 0 [0, 1], 67 | 0 [0, 2], 81 | 0.60 |
| Gravida | Median [IQR], n | 0 [0, 2], 67 | 1 [0, 3], 81 | 0.10 |
| **Fibroid assessment** | | | |  |
| Scanning modality to diagnose fibroid^b^ | MRI | 49 (73%) | 61 (75%) | 0.78 |
|  | Ultrasound | 17 (25%) | 19 (23%) |  |
|  | Not stated | 1 | 1 |  |
| Location of largest fibroid | Submucosal | 4 (6%) | 11 (14%) | 0.40 |
|  | Submucosal (pedunculated) | 0 (-) | 0 (-) |  |
|  | Subserosal | 13 (20%) | 14 (18%) |  |
|  | Subserosal (pedunculated) | 3 (5%) | 5 (7%) |  |
|  | Intramural | 45 (69%) | 46 (61%) |  |
|  | Other | 0 (-) | 0 (-) |  |
|  | Not stated | 2 | 5 |  |
| Longest dimension of largest fibroid, cm^a^ | <=7 | 32 (48%) | 43 (53%) | 0.52 |
|  | >7 | 35 (52%) | 38 (47%) |  |
|  | Mean (SD) | 8.04 (2.87), 67 | 7.57 (3.67), 81 | 0.38 |
| Number of fibroids^a^ | 1-3 | 43 (64%) | 53 (65%) | 0.82 |
|  | 4-10 | 21 (31%) | 26 (32%) |  |
|  | >10 | 3 (4%) | 2 (2%) |  |
|  | Median [IQR] | 2 [1, 5], 67 | 2 [1, 5], 81 | 0.72 |
| Largest fibroid volume, cm^3^ | Mean (SD), n | 457 (468), 65 | 466 (599), 80 | 0.92 |
| Uterine volume, cm^3^ | Mean (SD), n | 1238 (1262), 61 | 1174 (1216), 77 | 0.76 |
| **Medical and surgical history** | | | |  |
| Previous abdominal surgery^c^ | Caesarean section | 6 (9%) | 11 (14%) | 0.51 |
|  | Laparoscopy | 12 (18%) | 9 (11%) | 0.15 |
|  | Endometrial ablation | 2 (3%) | 2 (2%) | 0.37 |
|  | Appendectomy | 6 (9%) | 7 (9%) | 0.72 |
|  | Sterilization | 3 (4%) | 3 (4%) | 0.68 |
|  | Other | 6 (9%) | 14 (17%) | 0.14 |
| Taking contraceptive/ hormonal treatments to control symptoms, at randomization | | 43 (64%) | 48 (59%) | 0.45 |
| Footnote: ^a^ Minimisation variable; ^b^ More than one type of scan possible; ^c^ More than one previous abdominal surgery possible,  ^d^ T-test used for continuous data, chi-squared used for categorical. | | | | |

Table S 1 Baseline demographic, medical, surgical and fibroids characteristics of trial participants who responded to questionnaires at 4 years of follow-up.
